# Extracellular vesicles as vital players in drug delivery: a focus on clinical disease treatment

**DOI:** 10.3389/fbioe.2025.1600227

**Published:** 2025-05-14

**Authors:** Tiangang Chen, Dan Chen, Wanting Su, Jianlan Liang, Xiangning Liu, Mingxiang Cai

**Affiliations:** The First Affiliated Hospital of Jinan University, hospital of Stomatology, School of Stomatology, Clinical Research Platform for Interdiscipline of Stomatology, Jinan University, Guangzhou, China

**Keywords:** extracellular vesicles, drug delivery systems, separation and loading technology, clinical disease treatment, targeting

## Abstract

Extracellular vesicles (EVs), a diverse population of bilayer lipid-membrane vesicles secreted by cells, have emerged as ideal drug carriers due to their efficient cellular uptake and targeted delivery capabilities. Advancements in medical and bioengineering collaborations have enabled EVs to be engineered for specific marker expression or therapeutic cargo transport, positioning them as a promising modality for treating cancer, neurological disorders, cardiovascular diseases, and beyond. EV-based drug delivery strategies offer distinct advantages, including facilitation of intercellular communication and immune modulation, high biocompatibility and stability, the ability to traverse the blood-brain barrier, and potential synergistic interactions with encapsulated therapeutics to enhance efficacy. This review explores EV isolation and scalable production, emphasizing cost-effective and reproducible manufacturing strategies, cargo-loading methodologies, and therapeutic applications. Additionally, the current landscape of EV-based targeted drug delivery, clinical translation prospects, and prevailing challenges are examined to provide a comprehensive perspective on their potential in drug delivery systems.

## 1 Introduction

The existence of extracellular vesicles (EVs) was first reported as early as 1946, with De Broe later describing in 1977 that the release of these “cell membrane fragments” is a fundamental biological process in living cells. Initially, EVs were considered mere cellular waste disposal mechanisms, often likened to a “rubbish bag for the cell.” However, accumulating evidence has revealed their critical roles in various physiological and pathological processes, including immune modulation, neural communication, angiogenesis, and tissue repair, drawing significant research interest (Budnik et al., 2016; He et al., 2018).

EVs encompass a heterogeneous population of bilayer lipid-membrane vesicles secreted by nearly all cell types and detected in diverse body fluids. Based on differences in biogenesis and size, they are classified into three main subtypes: exosomes, microvesicles, and apoptotic vesicles (van Niel et al., 2018). These vesicles transport a diverse array of biomolecules—including proteins, nucleic acids, and lipids—modulating the cellular microenvironment via autocrine and paracrine signaling. Additionally, they circulate systemically, facilitating intercellular communication and molecular exchange at distant target organs (Sullivan et al., 2017), profoundly influencing both normal physiology and disease pathology (Stahl and Raposo, 2019). Due to their natural origin, EVs exhibit excellent biocompatibility, stability, and low immunogenicity. Their lipid bilayer confers protection against enzymatic degradation in bodily fluids and enables their passage across the blood-brain barrier (BBB) (Rufino-Ramos et al., 2017). These attributes position EVs as highly promising drug delivery vehicles. By engineering them to express specific surface markers or encapsulate therapeutic cargoes, they hold immense potential as platforms for treating cancer, neurological disorders, cardiovascular diseases, immune dysfunctions, and infectious diseases, offering novel opportunities for disease diagnosis and targeted therapy.

The intrinsic ability of EVs to transport bioactive molecules across cellular and biological barriers has established them as a rapidly emerging paradigm in drug delivery. Their superior delivery efficiency and modifiability provide a robust foundation for clinical applications. This review highlights recent advances in EV-based drug delivery systems, focusing on isolation and characterization techniques, cargo-loading strategies, and the therapeutic applications of EVs derived from different cell sources. Additionally, challenges in clinical translation and future research directions will be explored.

## 2 Extracellular vesicles

EVs are lipid bilayer vesicles secreted by nearly all cells and present in the body fluids of all organisms. Based on their biogenesis and size, EVs are classified into three main types: exosomes, microvesicles, and apoptotic vesicles (Kumar et al., 2024). These vesicles transport a broad array of biomolecules from their parent cells and play a pivotal role in cellular communication. Much like a fully loaded truck, the specific cargo carried by EVs determines their functional impact. The cargo primarily includes small and long molecules, coding and non-coding RNAs (e.g., mRNAs, miRNAs, lncRNAs), lipids, and proteins (Vader and Mol, 2016a). The biogenesis of EVs, influenced by the loading of different cargoes, contributes to their heterogeneity. Understanding the molecular mechanisms underlying biogenesis in different EV populations carrying diverse cargoes is essential for their role as intercellular communicators and therapeutic carriers (Dixson et al., 2023).

Recent advancements in EV research have solidified their significance as essential mediators of intercellular communication, with demonstrated diagnostic and therapeutic potential in cardiovascular disease, diabetes, and Alzheimer’s disease (AD). Their adaptability for targeted drug delivery further enhances their clinical utility (Couch et al., 2021). The unique properties of EVs confer distinct advantages as natural drug carriers. Notably, their ability to traverse biological barriers enables them to penetrate tissues and exploit endogenous cellular mechanisms for cargo sorting and delivery. Additionally, their intrinsic biocompatibility minimizes immune activation, mitigates toxicity associated with exogenous substances, and ensures structural stability in circulation, enhancing their safety profile (Elsharkasy et al., 2020). With advancements in bioengineering, EV targeting efficiency can be selectively optimized, making them an ideal vehicle for precision drug delivery (Vader and Mol, 2016a). EV-based delivery platforms are now employed for transporting siRNAs, miRNAs, proteins, small-molecule drugs, and nanoparticles, offering novel therapeutic strategies for various diseases (Meng et al., 2020).

## 3 Processes and mechanisms of extracellular vesicle biogenesis

EVs are categorized based on their size and biogenetic origin, encompassing exosomes (EXOs, 30–150 nm) derived from endosomes, microvesicles (MVs, 150–1,000 nm) budding from the plasma membrane, and apoptotic vesicles (200–5,000 nm) generated during apoptotic cell death (Wang et al., 2023). Given that apoptotic vesicles are rapidly recognized and phagocytosed by macrophages (Segawa and Nagata, 2015), and current EV-based drug delivery primarily targets EXOs and MVs, this review focuses on their biological functions and translational potential.

### 3.1 Biogenesis of extracellular vesicles

EV biogenesis is a complex process mediated by the interplay of various signaling and regulatory factors.

#### 3.1.1 Biogenesis of exosomes

**FIGURE 1:**
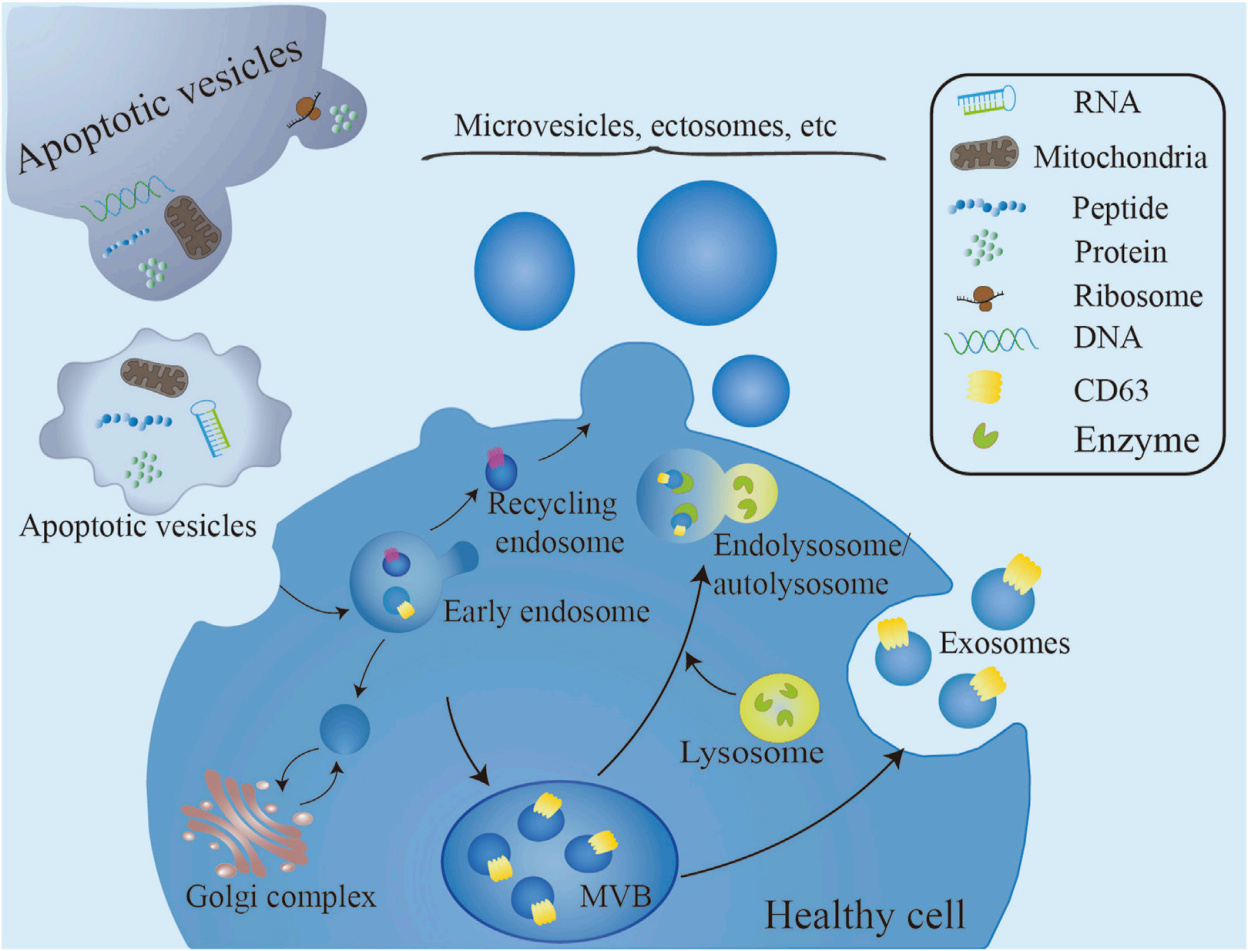
Exosome formation begins with the endocytic uptake of molecular cargo. Following cytophagy, endosomal membranes bud intracellularly to form intraluminal vesicles within the endosomal lumen. ILVs accumulate in multivesicular bodies, which can follow two distinct pathways: fusion with lysosomes for degradation or fusion with the plasma membrane for secretion into the extracellular space. A variety of extracellular vesicles, including microvesicles, bud directly from the cell surface. Biogenesis processes and mechanisms distinguish exosomes from microvesicles. Unlike exosome biogenesis, which begins with intracellular events, microvesicles are directly generated by outgrowth from the plasma membrane. Apoptotic vesicles, generated during apoptotic cell death under genetic regulation, contain biomaterials from the dying cell (Rilla, 2021).

Exosome formation begins with the endocytic uptake of molecular cargo (Krylova and Feng, 2023). The endocytic system comprises a network of dynamic membranous compartments essential for cargo sorting, processing, and degradation (Gould and Lippincott-Schwartz, 2009). Following endocytosis, the endosomal membrane buds inward to form intraluminal vesicles (ILVs), which later mature into exosomes. These early endosomes are characterized by cargoes destined for recycling, which localize to and detach from the peripheral tubular domains of the endosome. Upon detachment, these cargoes are transported to the Golgi network or plasma membrane for recycling. In contrast, unrecycled cargo accumulates in the central vesicular region of the early endosome, initiating the endosomal maturation pathway. This process leads to the transformation of early endosomes into late endosomes (Krylova and Feng, 2023), also referred to as multivesicular endosomes (MVEs) or multivesicular bodies (MVBs), due to the accumulation of multiple ILVs within their lumen. Subsequently, a subset of MVBs is directed to lysosomes or autophagosomes for degradation via fusion, while another subset is transported to the cytoplasmic membrane, where ILVs are released into the extracellular space through exocytosis (Raposo and Stoorvogel, 2013; Colombo et al., 2014) (Figure 1).

The regulation of ILV formation within endosomes involves multiple pathways and molecular mechanisms, broadly classified into ESCRT-dependent and ESCRT-independent processes (Hurley, 2015; Schöneberg et al., 2017; McCullough et al., 2018; Vietri et al., 2020; Krylova and Feng, 2023). The ESCRT (endosomal sorting complex required for transport) machinery comprises four distinct protein complexes (ESCRT-0, -I, -II, and -III) along with the AAA ATPase Vps4 complex (Henne et al., 2011; Henne et al., 2013). Functionally, the ESCRT system operates as a molecular framework for cargo selection and membrane remodeling. ESCRT-0, -I, and -II primarily mediate cargo recognition and sorting into specialized microdomains on the endosomal membrane, which serve as platforms for intracellular signaling. In contrast, ESCRT-III drives membrane invagination and scission, facilitating ILV formation. Following membrane cleavage, Vps4 promotes ESCRT-III disassembly, enabling recycling of its components. Beyond the ESCRT machinery, ILV biogenesis can also proceed via ESCRT-independent mechanisms. Various proteins and lipid constituents, including ceramides and tetraspanins, facilitate membrane invagination and vesicle formation in an ESCRT-independent manner.

#### 3.1.2 Biogenesis of microvesicles

Biogenesis processes and mechanisms distinguish exosomes from microvesicles. Unlike exosome biogenesis, which begins with intracellular events, microvesicles are directly generated by outgrowth from the plasma membrane (Figure 1) (Raposo and Stoorvogel, 2013; Clancy et al., 2021).

This process involves significant localized alterations in plasma membrane proteins and lipids, which modulate membrane curvature and stiffness (Kozlov et al., 2014; McMahon and Boucrot, 2015). The vertical redistribution of cargo components within the microvesicles complements the changes in the plasma membrane, leading to selective enrichment of certain molecules within the microvesicles. This unique mechanism of microvesicle formation ensures the regulated release of vesicles containing specific molecular cargo, a distinguishing feature compared to exosome biogenesis (Tricarico et al., 2016). Despite advances, the exact mechanisms of microvesicle formation and release remain incompletely understood. Current research primarily focuses on understanding membrane curvature and shape regulation in the context of phospholipid composition, lipid dynamics, and protein-mediated bending. For example, cholesterol plays a pivotal role in shaping nascent microvesicles, with depletion of plasma membrane cholesterol leading to reduced microvesicle production (Del Conde et al., 2005). Additionally, a combination of phospholipid redistribution and actin-mediated contractile mechanisms is critical to microvesicle biogenesis (Del Conde et al., 2005; Muralidharan-Chari et al., 2009). Notably, Shurer and colleagues (Shurer et al., 2019) found that long-chain biopolymers within the glycocalyx, which are anchored to the cell surface, play a critical role in regulating membrane curvature, potentially influencing microvesicle release.

### 3.2 Pathway for extracellular vesicle uptake by recipient cells

Upon release, EVs circulate in body fluids, reaching recipient cells to deliver their cargo, which triggers functional responses and promotes phenotypic changes. These changes may lead the recipient cell to secrete its own EVs. Depending on the cell type, EVs may remain attached to the plasma membrane, maintaining contact with receptors on the recipient cell surface to initiate intracellular signaling pathways (Buschow et al., 2009). Alternatively, EVs may be internalized by recipient cells through various mechanisms, including macropinocytosis, phagocytosis, and endocytosis via caveolae or lipid rafts (van Niel et al., 2018). The fate of the EVs depends on their specific composition and the receptor cell’s surface structure.

Once internalized, EVs typically follow the endocytic pathway, eventually reaching the MVE. Here, they often target lysosomes for degradation, providing relevant metabolites to the recipient cell (Zhao et al., 2016). However, internalized EVs can also avoid degradation through two alternative pathways. First, some vesicles escape degradation by fusing with the limiting membrane of the MVE, releasing their cargo into the cytoplasm of the recipient cell (Bissig and Gruenberg, 2014). Second, EVs may bypass lysosomal degradation via the early endocytosis-recycling pathway or undergo re-secretion through fusion of the MVE with the plasma membrane (Heusermann et al., 2016).

Current research identifies three main mechanisms by which EVs signal to recipient cells: (1) EVs bind to and activate receptors on the recipient cell membrane, initiating functional responses (Desrochers et al., 2016); (2) EVs are internalized and activate intracellular processes in recipient cells (Valadi et al., 2007; Skog et al., 2008); and (3) EVs fuse directly with the plasma or endosomal membrane of the recipient cell, facilitating the exchange of transmembrane proteins and lipids (Coleman and Hill, 2015).

This study aims to investigate the membrane interactions and intercellular fate of EVs. Although EV size and cargo may vary, the principles of uptake and intercellular transport across different EV subpopulations are likely similar (van Niel et al., 2018).

## 4 Extracellular vesicles as drug delivery vehicles

A drug delivery system refers to a formulation or device designed to enable the targeted and specific delivery of therapeutic agents to a designated site of action—such as cells, tissues, or organs—thereby exerting therapeutic effects (Sun et al., 2023). As previously noted, EVs are primarily composed of lipid bilayers, which encapsulate biologically active molecules, including nucleic acids, proteins, and metabolites. Their ability to selectively target specific cells, coupled with excellent biocompatibility and efficient drug-carrying capacity, makes EVs highly effective natural drug carriers. They are capable of encapsulating small drug molecules, RNAs, and proteins (Rädler et al., 2023). Furthermore, EVs are adept at crossing biological barriers—such as tissue, cellular, and intracellular barriers—thereby enhancing bioavailability by delivering drugs directly into target cells while shielding them from degradation (Elsharkasy, Nordin, Hagey, de Jong, et al., 2020). This is partly due to their intrinsic stability in the bloodstream, which is attributed to the negative charge on their surface and their ability to evade the mononuclear phagocytosis system (MPS) through the surface protein CD47 (de Jong et al., 2019). Given these remarkable properties, EVs are well-suited as drug delivery vehicles (Mulcahy et al., 2014; Costa Verdera et al., 2017; Joshi et al., 2020; O’Brien et al., 2022).

In conclusion, as drug carriers, EVs offer advantages including enhanced stability, improved water solubility, prolonged circulation time, sustained drug release, and stronger tissue/cellular targeting. Additionally, they reduce the required drug dose in clinical treatments, minimize side effects, and improve therapeutic efficacy.

## 5 Methods and techniques for isolating or preparing extracellular vesicles

**FIGURE 2:**
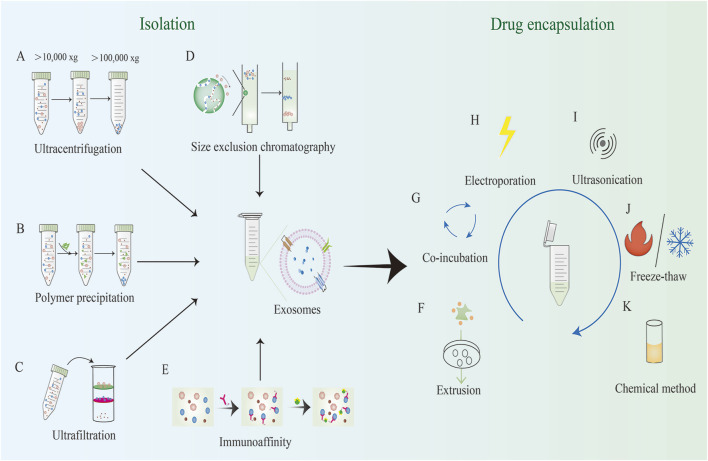
Common methods for EV isolation and exogenous loading. EV isolation methods include: **(A)** Ultracentrifugation; **(B)** Polymer precipitation; **(C)** Ultrafiltration; **(D)** Size exclusion chromatography; **(E)** Immunoaffinity, etc. Exogenous loading involves the direct introduction of drugs or therapeutic molecules into pre-isolated EVs through physical, chemical, or biological methods. Exogenous loading methods include: **(F)** Extrusion; **(G)** Co-incubation; **(H)** Electroporation; **(I)** Ultrasonication; **(J)** Freeze-thaw; **(K)** Chemical methods, etc.

Efficient isolation of EVs is crucial for subsequent research and applications. This paper reviews the commonly used EV isolation methods (Figure 2), highlighting their advantages and disadvantages.

Table 1 compares the common EV isolation methods, detailing their respective advantages and limitations.

**TABLE 1:** Comparison of common methods for isolating EVs and their advantages and drawbacks.

Methodologies	Mechanisms	Advantages	Drawbacks	Ref
UC	Separation by differential centrifugation	Low protein contamination	1. Expensive 2. Low flux 3. May damage EVs or induce extracellular vesicle aggregation 4. Unable to separate similarly sized particles	Théry et al. (2006), Linares et al. (2015a), Coumans et al. (2017a), Gandham et al. (2020)
Ultrafiltration	Use of ultrafiltration membranes with specific pore sizes	Simple, time-saving, and relatively gentle	1. Alter the structural integrity of the EVs 2. May clog pores or adherence	Taylor and Shah (2015a), Visan et al. (2022), Brezgin et al. (2024)
SEC	Fluid dynamics differences	Low impurities and high purity	1. Low resolution of EVs isolates 2. EVs are diluted	Baranyai et al. (2015), Lobb et al. (2015), Taylor and Shah (2015a), Takov et al., 2019; Gandham et al. (2020)
Polymer precipitation	The polymer can form a hydrophobic microenvironment around EVs and induce reversible EV aggregation	Simple, fast, and can handle large numbers of samples	1. Low EVs purity 2. Higher levels of heterogeneous proteins 3. Difficult to remove polymer	Konoshenko et al. (2018a), Colao et al. (2018)
Immunoaffinity	Antigen-antibody-specific recognition and binding	High specificity	1. High cost 2. Non-specific binding, competitive inhibition, and antibody cross-reactivity	Li et al. (2017), Konoshenko et al. (2018b), Visan et al. (2023)

### 5.1 Ultracentrifugation

Ultracentrifugation (UC) remains the “gold standard” for isolating EVs, leveraging differences in particle size and density to separate them from other cellular components. The process begins with low-speed centrifugation to remove cells and debris, followed by high-speed centrifugation at ≥100,000 g to isolate EVs with minimal protein contamination (Théry et al., 2006).

Despite its widespread use, UC has notable limitations: it has low throughput, requires expensive and specialized equipment, and may cause EV membrane damage or aggregation during the process (Linares et al., 2015a; Coumans et al., 2017a). Additionally, blood samples often contain components of similar size and density to EVs, such as lipoproteins and cellular particles, which cannot be fully removed by UC(Gandham et al., 2020).

### 5.2 Ultrafiltration

Ultrafiltration offers an alternative approach, using membranes with varying molecular weight cut-offs to selectively separate EVs. Compared to UC, ultrafiltration is simpler, more efficient, and preserves the biological activity of EVs. However, the method can suffer from membrane blockage due to the filtration of small molecules (Taylor and Shah, 2015a). To address this, tangential flow filtration (TFF) technology has been developed to prevent membrane surface deposits, thus minimizing the risk of pore blockage and surface contamination (Visan et al., 2022; Brezgin et al., 2024). Nevertheless, filtration may still impact EV structural integrity due to shear forces, potentially causing the loss of EVs.

### 5.3 Size exclusion chromatography

Size exclusion chromatography (SEC) separates sample components based on hydrodynamic volume, with small molecules entering more pores and eluting later, while larger molecules elute earlier (Taylor and Shah, 2015a). SEC is an effective, straightforward, and robust method that requires no expensive equipment. Typically performed using Sepharose CL-2B or similar columns under mild conditions, SEC can achieve high-purity separation of exosomes from plasma with minimal impurities (Baranyai et al., 2015; Lobb et al., 2015; Takov et al., 2019). However, SEC suffers from low resolution in separation and dilution limitations (Gandham et al., 2020).

### 5.4 Polymer precipitation

The polymer precipitation method employs polymers to create a hydrophobic microenvironment around EVs, promoting aggregation and enabling their separation via low-speed centrifugation. Commonly used polymers include hydrophilic substances like polyethylene glycol (PEG), fish protein, and sodium acetate (Konoshenko et al., 2018a). This method, second only to UC in popularity for EV isolation, allows high-throughput processing of samples, is simple and rapid, and does not deform the EVs (Konoshenko et al., 2018a). However, it often leads to the introduction of foreign proteins, resulting in lower EV purity, and generates residual polymers that are difficult to remove. Therefore, additional purification steps are necessary to enhance the purity of the isolated EVs (Colao et al., 2018).

### 5.5 Immunoaffinity

Immunoaffinity methods exploit antigen-antibody-specific recognition and binding to isolate EVs. Common techniques include flow-through, magnetic bead, and kit-based approaches. In these methods, specific antibodies are used to couple with the surface molecules of EVs (such as lipids, proteins, and polysaccharides), enabling the capture of EVs with high purity and minimizing contamination from soluble proteins. The process is relatively simple and preserves the morphological integrity of the exosomes. However, these methods can be expensive, and EVs are often difficult to elute from the antibody complexes. Furthermore, issues like nonspecific binding, competitive inhibition, and antibody cross-reactivity can reduce separation efficiency (Konoshenko et al., 2018a).

## 6 Methods and techniques for loading cargo in extracellular vesicles as drug delivery systems

EVs are considered one of the most promising natural carriers for drug delivery systems due to their ability to effectively cross biological barriers—such as tissue, cellular, and intracellular barriers—along with their inherent targeting properties, utilization of natural intracellular transport pathways, and high biocompatibility (Elsharkasy, Nordin, Hagey, De Jong, et al., 2020). This section outlines two distinct methods for loading cargo into EVs: exogenous loading (post-separation) and endogenous loading (pre-separation) (Vader and Mol, 2016a).

### 6.1 Endogenous loading

Endogenous loading refers to the integration of substances, such as RNA, proteins, and small molecules, into EVs during their biogenesis. This method preserves the integrity of the EV membrane (Walker et al., 2019). Common endogenous loading techniques include transfection and co-incubation.

#### 6.1.1 Transfection

Transfection-based methods are often used for loading small RNAs into EVs, capitalizing on EVs’ natural role as carriers of small RNAs. In this process, transfection reagents are used to introduce miRNA, siRNA, or plasmid DNA (pDNA) into donor cells, inducing overexpression of a target gene. The gene product is then encapsulated within EVs, which are actively secreted as biogenesis proceeds (Kosaka et al., 2010; Lou et al., 2015; Darband et al., 2018). Some studies have indicated that the type and/or sequence of RNA may influence the efficiency of RNA integration into EVs (Koppers-Lalic et al., 2014). However, this method may also lead to the incorporation of unwanted proteins, RNAs, or other cellular components, necessitating the development of new technologies to eliminate these undesired molecules or prevent their inclusion in exosomes (Brezgin et al., 2024).

#### 6.1.2 Co-incubation

In co-incubation, donor cells are incubated with therapeutic agents, allowing these drugs to be naturally encapsulated within EVs. For instance, one study demonstrated that mesenchymal stem cells (MSCs) incubated with high doses of paclitaxel successfully loaded paclitaxel into EVs, which effectively inhibited tumor growth and angiogenesis *in vitro* (Pessina et al., 2011). Similarly, incubating hepatocellular carcinoma cells with chemotherapeutic agents such as paclitaxel, carboplatin, and etoposide allowed these drugs to be encapsulated within EVs, enhancing their cytotoxicity against natural killer cells (Lv et al., 2012).

### 6.2 Exogenous loading

Exogenous loading involves the direct introduction of drugs or therapeutic molecules into pre-isolated EVs through physical, chemical, or biological methods. Also known as direct loading or post-loading, this approach allows for a broader range of therapeutic agents to be loaded into EVs compared to endogenous loading (Figure 2).

#### 6.2.1 Exosome electroporation

Given that EVs share the same phospholipid membrane structure as cell membranes, hydrophilic pores can be temporarily formed in the EV membrane under pulsed current, facilitating the entry of hydrophilic small molecules and nucleic acids into EVs (Chen et al., 2024). Several studies have demonstrated the successful loading of siRNA into EVs via electroporation, with EV products subsequently delivered to target organs (Greco et al., 2016; Lunavat et al., 2016). Electroporation is favored due to its ease of operation, efficiency, speed, and controllable parameters, making it a widely used loading method (Familtseva et al., 2019). However, while electroporation improves drug loading, it can also induce RNA precipitation, alter EV membrane morphology, and cause aggregation (Kooijmans, 2013; Johnsen et al., 2016). Nevertheless, optimized electroporation parameters can maintain EV membrane integrity and preserve cargo activity.

#### 6.2.2 Co-incubation

Co-incubation involves incubating the isolated EVs with the target drug or bioactive molecule, followed by purification through UC or filtration. This method is the simplest, most cost-effective, and best preserves the integrity of the EV membrane. It is particularly suitable for small-to medium-sized hydrophobic molecules and membrane-permeable drugs, such as curcumin, dopamine, and celastrol (Zhang et al., 2019; Chen et al., 2024). However, its efficiency in drug loading is generally lower compared to other methods.

#### 6.2.3 Ultrasonic loading method

Ultrasonication utilizes probe ultrasound to generate transient pores in the EV membrane, enhancing membrane permeability and allowing the diffusion of small hydrophilic molecules into the EVs. This method is similar to electroporation and may also cause damage to the EV membrane (Antimisiaris et al., 2018). However, it has been shown that incubating EVs at 37°C for 1 hour can restore membrane integrity (Kim et al., 2016), with minimal impact on lipid content or membrane-bound proteins, preserving the overall structure of the exosomes.

#### 6.2.4 Extrusion

Extrusion involves the combination of a drug solution with an EV suspension, which is then loaded into a lipid extruder equipped with a 100–400 nm porous membrane syringe. The drug is introduced into the EVs through shear mechanical forces under controlled temperature conditions (Luan et al., 2017). However, extrusion can lead to re-formation or deformation of exosomes. For example, multiple extrusion cycles (31) of porphyrin-loaded EVs derived from MDA-MB231 breast cancer cells altered the size distribution and zeta potential of the original EVs, resulting in cytotoxicity (Fuhrmann et al., 2015). The precise effects of extrusion on the membrane structure and properties remain unclear.

#### 6.2.5 Thawing and freezing

The freeze-thaw method involves mixing the cargo with EVs, followed by rapid freezing in liquid nitrogen and thawing at room temperature. This cycle is repeated several times, utilizing heat to promote drug loading into the EVs (Walker et al., 2019). Similar to other techniques, freeze-thaw methods can induce EV aggregation and alter membrane properties (Haney et al., 2016).

#### 6.2.6 Chemical method

Chemical methods rely on chemicals to increase the permeability of EV membranes for cargo loading. Common approaches include chemical permeation, pH gradients, low permeability dialysis, and lipid binding (Brezgin et al., 2024). Chemical permeability typically utilizes saponins, which bind to cholesterol in the EV membrane, forming pores that facilitate drug entry (Peng, 2020). However, saponins can damage the EV membrane, alter the zeta potential, and exhibit toxic and hemolytic activity *in vivo*, which limits their use in drug loading (Peng, 2020; Hettich et al., 2022; Dimik, 2023).

## 7 Therapeutic applications of extracellular vesicles as drug carriers

EVs play a critical role in cellular communication by efficiently transporting their cargo to both neighboring and distant cells, regulating various pathological processes. Due to their excellent biocompatibility, low immunogenicity, and ability to facilitate intercellular signaling, EVs have emerged as promising carriers for treating a wide range of diseases. In regenerative medicine, EV-based nano-therapy for delivering nucleic acids, proteins, and drugs to injury sites has gained significant attention. This section will focus on the application of EVs as drug delivery vehicles in treating diseases, especially neurodegenerative diseases, cancer, and respiratory conditions.

### 7.1 Neurodegenerative diseases

Treating neurological disorders, such as Parkinson’s disease (PD), AD, and stroke, is challenging due to the inefficiency of drug delivery across the BBB, a selective barrier that prevents most macromolecules from entering the brain, allowing only small molecules to pass from the bloodstream (Abbott et al., 2010). Many therapeutic agents struggle to penetrate the BBB effectively, resulting in low drug bioavailability at the target site (Shahjin et al., 2020). Recently, nanomedicine systems leveraging the ability of nanovesicles to cross the BBB have garnered significant attention for treating neurological conditions (Nemeth et al., 2019). EVs are capable of delivering drugs, proteins, mRNAs, and other nucleic acids efficiently, making them ideal drug carriers with the potential to slow or halt the progression of neurological diseases (Peng et al., 2022).

AD, a prevalent neurodegenerative condition, is clinically characterized by memory loss and cognitive decline (Hampel et al., 2018). A hallmark of AD pathology is the accumulation of β-amyloid peptide (Aβ) in amyloid plaques (Cummings et al., 2019).

EEV-mediated delivery of therapeutic agents to the brain can modulate proteins linked to Aβ deposition and neuroinflammation, thereby intervening in key pathological processes of AD. Specifically, one study encapsulated miRNA-29 in MSC-derived exosomes (MSCs-Exo), which were locally injected into AD model rats, resulting in modulation of proteins related to β-amyloid deposition and neuroinflammation, leading to significant cognitive function improvement (Jahangard et al., 2020). One study evaluated the natural ability of various exosomes to cross the blood-brain barrier (BBB) and found that all tested exosomes could traverse the BBB, with parenchymal uptake rates ranging from 58% to 93%. This suggests that exosomes from diverse sources appear capable of crossing the BBB and entering the brain; however, their ability to access target brain regions exhibits significant heterogeneity (Banks et al., 2020).

In stroke therapy, EVs loaded with nerve growth factor (NGF) (e.g., RVG-EV) notably enhance NGF expression in the infarcted region, reduce inflammation, and promote cell survival (Yang et al., 2020).

In a study of PD, Haney et al. employed peroxidase-loaded macrophage exosomes in a PD mouse model, demonstrating that these drug-loaded exosomes not only preserved peroxidase activity and extended drug circulation time but also effectively reduced immunogenicity, thereby enhancing therapeutic efficacy (Haney et al., 2015). Exosomes have also been utilized to deliver anti-inflammatory molecules to suppress hyper-immune and inflammatory responses following spinal cord injury (SCI). For instance, M2 macrophage-derived exosomes loaded with berberine (BER) significantly mitigated inflammation and promoted motor function recovery after SCI (Gao et al., 2021).

These findings highlight the ability of EVs to transport various therapeutic agents—drugs, genes, and proteins—across the blood-brain barrier, offering a synergistic approach to treating neurological disorders and presenting a novel strategy for disease-targeted therapies.

### 7.2 Cancer

Cancer remains a significant threat to human health and is a leading public health concern globally (Siegel et al., 2023). Conventional anticancer drugs often face challenges such as poor stability, off-target effects, and undesirable side effects, limiting their clinical effectiveness (Kumar et al., 2023). Consequently, there is a pressing need to develop novel drug delivery systems to improve drug stability, enhance targeting, and minimize harm to healthy tissues. Recent research has explored exosomes from various cellular origins for treating diverse cancers, including oral leukoplakia, triple-negative breast cancer, and colon adenocarcinoma. Efforts are underway to engineer more efficient drug delivery systems through genetic modification and cargo-loading technologies to amplify therapeutic efficacy (Chulpanova et al., 2021; Weaver et al., 2022; Klimova et al., 2024).

EVs have gained widespread attention in preclinical studies for delivering chemotherapeutic agents, such as paclitaxel, cisplatin, and doxorubicin (DOX), to improve drug targeting while minimizing side effects on healthy tissues. By directly targeting cancer cells, these EV-based systems reduce systemic toxicity and elevate local drug concentrations (Bagheri et al., 2020; D’Agnano and Berardi, 2020). Drug-loaded EVs can precisely target diseased tissues, exhibiting enhanced efficacy and reduced off-target effects compared to free drugs. This approach has been successfully demonstrated in recent studies using MSC-derived EVs to enhance DOX uptake, augment its antitumor effects, and minimize toxicity to other organs. The anticancer effects of DOX-loaded EVs were shown to surpass those of free DOX (Schindler et al., 2019; Wei et al., 2022). This delivery system has proven effective across various cancers, including breast, pancreatic, and liver cancers (Song et al., 2021; Bi et al., 2024). For example, BI et al. developed a novel nanodrug carrier (EVPA) by encapsulating paclitaxel (PTX) into EVs via physical extrusion. The results demonstrated that the EVPA group significantly reduced tumor growth rate, with tumor size remaining below 40 mm^3^ (Bi et al., 2024). In the study by Li et al., although a high-dose doxorubicin (DOX) treatment in a mouse lung cancer model exhibited tumor growth inhibition, the mice suffered severe body weight loss and died by day 10. In contrast, co-loading doxorubicin (DOX) and lapatinib (LND) into EVs for delivery (DOX/LND-EVs group) showed more pronounced tumor suppression, with relative tumor volume and tumor weight reduced by 53% and 47%, respectively, and no cardiotoxic damage was observed in the mice (Li et al., 2022).Similarly, in a recent study, researchers led by W. Xu developed an Exo-Dox-NP delivery system for a mouse orthotopic breast cancer model. The results showed that the tumor weight in the control group was more than three times that of the Exo-Dox-NP group, indicating a significant enhancement in the anticancer efficacy of DOX without adverse reactions. *In vitro* experiments demonstrated a significantly enhanced inhibition of breast cancer cell migration and invasion, surpassing the inhibitory effects of free Dox. This may be attributed to the slow release of Dox and the structural stability of Exos as a carrier, which provided significant advantages (Xu, 2024a). These findings are consistent with the discoveries by Gomari et al. and Wei et al. (Gomari et al., 2019; Wei et al., 2019), suggesting that exosomes can serve as promising candidates for drug delivery vehicles.

In addition to chemotherapeutics, EVs have also emerged as powerful delivery vehicles for gene therapy and immunotherapy. They can transport siRNAs, miRNAs, or gene-editing tools, such as CRISPR/Cas9 systems, into target cells for gene-level intervention (Roerig et al., 2022; Corvigno et al., 2024). For instance, REV has been shown to inhibit tumor progression in breast cancer xenograft models by delivering antisense oligonucleotides (ASOs) without inducing systemic inflammation or toxicity (Xu et al., 2021). Moreover, studies have demonstrated that siRNA targeting DARS-AS1 encapsulated in EVs can effectively silence tumor-associated genes in TNBC cells, suppressing metastasis induced by chronic unpredictable mild stress, and inhibiting tumor growth and invasion (Liu et al., 2022).

As an innovative nanocarrier, EVs not only protect drugs from environmental degradation but also deliver them directly to tumor sites, reducing damage to healthy tissue. This capability holds significant promise for cancer treatment, offering a potential solution for targeted drug delivery and personalized therapeutic approaches.

### 7.3 Diseases of the respiratory system

With the growing interest in EVs for cancer therapy, researchers are increasingly investigating their potential as nanocarriers in the treatment of respiratory diseases (Gao et al., 2023). Popowski et al. explored the feasibility of lung-derived exosomes (Lung-exo) as an inhalation drug delivery system, demonstrating that Lung-exo efficiently delivered mRNA and protein drugs to the bronchioles and alveoli after inhalation. These exosomes also exhibited high retention in lung tissue, significantly enhancing the bioavailability of these drugs in the lungs (Popowski et al., 2022). Another study utilized co-incubation to load Glycyrrhetinic acid (GA) into milk-derived extracellular vesicles (mEVs@GA) for direct delivery to the lungs via inhalation. EV-mediated delivery of GA showed potent anti-inflammatory and anti-fibrotic effects in the lungs, while minimizing systemic side effects, providing a promising new approach for treating idiopathic pulmonary fibrosis (Ran et al., 2024). In sepsis, the over-activation of PD-1 leads to immunosuppression, impairing immune response and worsening lung damage. An exosome-based system that delivers anti-PD-1 peptides, via human umbilical cord mesenchymal stem cell exosomes, plays a critical role in treating sepsis-induced acute lung injury by precisely targeting immune cells or damaged tissues (Huang, 2024a). These studies underscore the considerable potential of EVs as carriers for lung-targeted therapy, immunomodulation, and anti-inflammatory treatments.

### 7.4 Skin diseases

In addition to respiratory diseases, EVs have shown promise in skin repair, offering a scientific basis for their use in treating skin conditions (Zhang et al., 2016).

MiR-155, which is involved in various inflammatory and immune responses, has been linked to delayed wound healing in diabetes. When Gondaliya et al. loaded miR-155 inhibitors into exosomes, mice in the experimental group exhibited significantly increased collagen deposition, angiogenesis, and reepithelialization, accelerating wound healing and promoting more effective tissue regeneration (Gondaliya et al., 2022). Moreover, melatonin-loaded exosome-mimicking nanovesicles administered locally in an atopic dermatitis model significantly improved skin lesions, alleviating swelling and pruritus and restoring skin barrier function (Kim et al., 2021). These findings highlight the vast potential and therapeutic applications of EVs in skin disease treatment.

### 7.5 Bone-related diseases

Cell-derived EVs have gained significant attention in the treatment of various bone-related diseases, particularly for their roles in alleviating inflammation, promoting bone repair, and enhancing bone density (Behera et al., 2021; Torrecillas-Baena et al., 2023). To improve therapeutic outcomes, researchers continue to optimize the structure of EVs, enhancing their drug-carrying capacity and targeting properties to increase efficacy while minimizing side effects (Kang et al., 2022; Sadeghian-Nodoushan et al., 2022).

Targeted bone delivery using milk-derived exosomes (MDEVs), coupled with MRI monitoring, has paved the way for personalized and precise treatment strategies for osteoporosis. Researchers modified bone-targeting peptides on the surface of exosomes loaded with both SRT2104 and an MRI contrast agent. This engineered (DSS)_6_-mEV-SRT2104 not only exhibited the anti-osteoporotic properties of both mEVs and SRT2104 but also incorporated MnB nanoparticles for MRI visualization *in vivo*. In an ovariectomized (OVX) mouse model, these MRI-assisted engineered mEVs demonstrated promising therapeutic effects by inhibiting bone resorption and stimulating bone formation (Huang, 2024b).

Surface-modified exosomes loaded with miR-140 and chondrocyte-targeting peptides have also shown significant potential in slowing the progression of osteoarthritis in rats (Liang et al., 2020). In another study, magnetic nanoparticles (GMNPs) were combined with BMSC-derived exosomes to deliver MEG3 and regulate miR-3064-5P expression. Overexpression of MEG3 inhibited miR-3064-5p function, inducing mitophagy in rats to improve bone metabolism and alleviate diabetes-induced osteoporosis (Xu, 2024b). Additionally, transfection of miRNA-375 into human adipose mesenchymal stem cells resulted in miRNA-375-enriched exosomes, which were effective in treating bone defects in mice by promoting osteoblast proliferation, differentiation, and bone regeneration (Chen et al., 2019).

Surface modification with targeted peptides or small molecules allows for precise targeting of specific bone tissues, enhancing drug concentration at the target site and minimizing systemic side effects. Furthermore, real-time dynamic monitoring, in conjunction with imaging technologies like magnetic resonance imaging (MRI), enables optimization of treatment plans, evaluation of efficacy, and personalized adjustments, ensuring more precise and efficient therapies.

### 7.6 Eye diseases

EVs loaded with conventional anti-inflammatory drugs have been explored for the treatment of ocular diseases. A study demonstrated the enhanced therapeutic effect of milk-derived exosomes loaded with curcumin in reducing retinal pigment epithelial cell damage (Wu et al., 2025). Curcumin, a natural compound known for its anti-inflammatory and antioxidant properties, has limited bioavailability when administered conventionally (Li et al., 2021). By encapsulating curcumin in milk-derived exosomes, researchers successfully enhanced its stability and bioavailability, thereby more effectively mitigating retinal pigment epithelial cell damage. Another study examined the anti-inflammatory and reparative effects of milk-derived exosomes loaded with dexamethasone (DEX) in a corneal alkali burn model, further highlighting the therapeutic potential of EVs as drug delivery systems in ocular diseases (Wang et al., 2024). These findings suggest that EVs can serve as an effective platform for improving drug bioavailability and enhancing therapeutic efficacy in eye treatments.

### 7.7 Gastrointestinal diseases

The gastrointestinal (GI) environment presents unique challenges for drug delivery systems, including its high acidity, digestive enzymes, and rapid transport characteristics. Current research on gastrointestinal diseases primarily focuses on EVs derived from milk and plants (Zeng et al., 2024), which offer significant advantages in oral drug delivery (Xiao et al., 2022).

For instance, ginger-derived exosomes loaded with TNF-α siRNA and ZIF-8 nanocarriers have demonstrated significant immunomodulatory effects by targeting and silencing TNF-α genes, suppressing inflammation, and modulating the gut microbiota to reduce inflammation in ulcerative colitis (Cui et al., 2024). Another study found that Sophora flavescens-derived exosome-like nanovesicles loaded with CX5461 effectively alleviated DSS-induced colitis in mice, highlighting the potential of natural-derived nanocapsules to improve drug bioavailability and therapeutic efficacy, offering a novel strategy for inflammatory bowel disease treatment (Zhang et al., 2024). Additionally, milk-derived exosomes have been utilized to bind anti-TNF-α nanoantibodies and antimicrobial peptides, reducing inflammation and improving the gut microbial environment (Jing et al., 2024).

Despite these advances, challenges remain in gastrointestinal drug delivery systems, particularly due to individual differences such as variations in gastrointestinal pH and enzyme activity, which can affect the effectiveness of oral drug delivery. Ensuring the stability of drugs during oral administration and their controlled release at the target site remains a key challenge in research and development. Recently, MSC-derived exosomes loaded with BER have been administered intravenously to a mouse model of ulcerative colitis, increasing the bioavailability of BER and enhancing its anti-inflammatory effects. This approach provides a new route of administration for treating gastrointestinal disorders (Deng et al., 2024), offering additional therapeutic options for patients whose oral medications are not effectively absorbed.

### 7.8 Cardiovascular diseases

Cardiovascular diseases, including coronary artery disease, hypertension, myocardial infarction, congenital heart defects, and peripheral vascular diseases, pose significant health risks globally (Davidson et al., 2021). In cardiovascular disease, EVs not only serve as biomarkers for disease progression but also act as vehicles for delivering therapeutic agents directly to damaged cardiovascular tissues (Liu et al., 2022). Through engineering modifications, EVs can be loaded with specific therapeutic molecules, such as anti-inflammatory or antithrombotic drugs, to enhance their therapeutic efficacy (Tong et al., 2025).

For instance, Sruti et al. encapsulated endothelial-specific miR-126 into CKIT + progenitor (CPC)-derived EVs via electroporation. These modified EVs, carrying custom cargo while maintaining their membrane structure for cellular uptake, enhanced myocardial repair after ischemia-reperfusion (I/R) injury (Bheri et al., 2023). In several other studies, adipose-derived stem cells (ADSCs) overexpressing miR-126 and EVs secreted by HEK293T cells were shown to promote microangiogenesis, migration, reduce cardiac fibrosis, and enhance anti-apoptotic effects. These genetically engineered EVs offer promising strategies for treating myocardial infarction (MI) and could have significant clinical implications in the future (Luo et al., 2017; Song et al., 2019).

Additionally, peptide-functionalized milk-derived exosomes have been shown to deliver miR-30d to the heart, improving cardiac hypertrophy, fibrosis, and overall cardiac function (Tong et al., 2025).

### 7.9 Kidney diseases

Drug delivery to the kidney is complicated by its complex structure and the glomerular filtration barrier. DEX, an effective anti-inflammatory agent for various kidney diseases, has limited clinical use due to severe adverse reactions. By constructing M2 macrophage-derived EVs loaded with DEX, researchers found that these EVs targeted injured kidneys, exerted anti-inflammatory and anti-fibrotic effects, and significantly reduced the adverse effects of DEX on blood glucose levels and the hypothalamic-pituitary-adrenal axis in mice (Tang et al., 2019).

Tang et al. used macrophage-derived EVs as a vector to deliver interleukin-10 (IL-10) to alleviate acute kidney injury (AKI). This approach significantly ameliorated I/R-induced kidney injury and reduced chronic lesions by enhancing the stability and targeted delivery of IL-10 to the kidney (Tang et al., 2020). In another study, erythrocyte-derived EVs were modified with a renal injury molecule-1-targeting peptide (LTH) to deliver siRNAs targeting P65 and SNAI1. This siRNA delivery system successfully delivered the therapeutic molecules to injured renal tubules, effectively reducing renal tubulointerstitial inflammation and fibrosis induced by I/R and unilateral ureteral obstruction, thus preventing the chronic progression of AKI (Tang et al., 2021). These studies highlight the potential of EVs as a powerful tool for delivering various therapeutic agents, including nucleic acids, proteins, and small molecules, in the treatment of renal diseases.

### 7.10 Infectious diseases

Many anti-infective drugs, including antibiotics and antiviral agents, suffer from poor oral absorption and short half-lives, limiting their therapeutic efficacy (Murray et al., 2022; Bizzarri et al., 2024). With the rising challenges of antibiotic and antiviral resistance, researchers are exploring innovative drug delivery techniques to enhance the effectiveness of these drugs.

Recent studies have demonstrated the potential of exosomes as delivery vehicles. For example, exosomes expressing the HIV-1-specific antibody scFv, loaded with either curcumin or miR-143, showed the ability to target Env + cells and tissues, inhibiting tumor growth and inducing targeted cell death (Zou et al., 2019). In another study, engineered EVs were used to deliver CRISPR-Cas9 ribonucleoproteins (RNPs), significantly inhibiting HSV-1 replication. The packaging efficiency of Cas9 RNP in EVs was enhanced using the Fc/Spa interaction system, offering a promising approach for viral gene editing (Wan et al., 2024).This study demonstrated that engineered EVs exhibit therapeutic potential in both *in vitro* and *in vivo* experimental models; however, RVG-modified EVs exhibited reduced neuro-targeting efficiency *in vivo* compared to *in vitro* settings. The authors hypothesized that this discrepancy may arise from either BBB impediment or macrophage-mediated endocytosis under physiological conditions. Regarding immunogenicity risk, multiple experimental datasets indicate that this engineered EV system demonstrated favorable safety profiles, including absence of cellular toxicity, unchanged body weights and no lesions in the organs of experimental mice, with non-immunogenic characteristics (attributed to the small peptide size of 29 aa).

However, the immune status, disease severity, and infection type influence the efficacy of drug delivery systems, posing challenges in optimizing treatment regimens based on individual patient profiles.

### 7.11 Genetic diseases

Genetic diseases, caused by gene mutations, present additional challenges for traditional therapies, particularly in gene delivery and repair efficiency. With advancements in gene therapy, EVs have emerged as a promising delivery system due to their ability to carry genetic information effectively and target specific cells (Munagala et al., 2021). The delivery of biomolecular cargo such as mRNA, miRNA, and proteins via EVs can modify the genetic profiles and biological responses of recipient cells, offering new avenues for disease regulation (Marcus and Leonard, 2013).

For instance, in Huntington’s Disease (HD), an inherited neurodegenerative disorder caused by mutations in the Huntington gene (HTT), exosome-mediated delivery of siRNAs effectively silenced the Huntington gene, improving motor function and reducing neuropathological changes. Additionally, hydrophobically modified siRNAs delivered via exosomes not only enhanced siRNA stability but also improved endocytosis efficiency, providing a promising strategy for treating HD (Didiot et al., 2016).

These studies collectively demonstrate the successful loading of a diverse range of therapeutic agents—nucleic acids, proteins, and small molecules—into EVs. Through their natural biocompatibility and engineered modifications, EVs are emerging as a promising drug delivery platform for a wide variety of diseases. By improving drug targeting and efficacy, EVs not only enhance therapeutic outcomes but also minimize side effects, highlighting their potential in precision therapy.

## 8 Challenges of extracellular vesicles as drug delivery vehicles

EVs offer significant promise as drug delivery platforms, particularly for their efficiency in crossing the blood-brain barrier and their ability to carry various therapeutic cargoes (Simon et al., 2020). However, their clinical translation faces several substantial hurdles, including issues related to extraction purity, large-scale production, safety assessment, and regulation and ethics. Many clinical studies remain in the early stages, and widespread application of EV-based drug delivery systems is still a distant goal.

### 8.1 Production aspects

One of the primary challenges is the variability in EV isolation and purification methods, which can greatly influence the purity of the final product. Additionally, existing large-scale production techniques are not standardized, hindering the widespread adoption of EVs as therapeutic tools. Researchers have developed several new methods to address these challenges, such as optimizing donor cells, using bioreactors, and incorporating culture media additives to improve EV yield and quality (Bost et al., 2022). For example, manipulating MSCs or altering their culture conditions can increase EV production, but these approaches may inadvertently promote the generation of unhealthy cells, which can compromise the quality of EVs and introduce safety risks (Rezaie et al., 2022). Mendt et al. utilized GMP standards to produce clinical-grade exosomes in large-scale bioreactors (Mendt et al., 2018). However, this process requires precise control and quality management to ensure consistency and safety. Thus, developing novel production technologies that enable the large-scale production of high-purity exosomes while preserving their biophysical properties and structural integrity remains an urgent need.

Another challenge lies in the lack of a standardized protocol for drug encapsulation within EVs. While techniques such as extrusion, electroporation, freeze-thawing, and incubation have been employed for drug loading, each method presents limitations—such as structural disruption of EVs, loss of bioactivity, and low loading efficiency (Lu et al., 2024). Choosing the appropriate method depends on the specific needs of the application, and combining multiple approaches can often overcome individual drawbacks, improving the overall drug delivery efficiency.

In summary, while EV-based drug delivery systems hold immense potential, significant challenges related to standardization, production scalability, and drug encapsulation remain. Rigorous characterization of EVs is crucial to ensure batch-to-batch consistency and maintain the safety and efficacy of the final product.

### 8.2 Cell source aspects

EVs carry cell-specific and parent cell-unique biomolecules that are functionally linked to the parent cell. Given that the function of EVs is closely tied to that of their source cells (Fernández-Rhodes et al., 2024), different types of EVs may be more suitable for specific disease therapies. Various factors influence the selection of an appropriate EV source, and to ensure their safety in clinical applications, strict control over their origin—such as species, cell type, and culture conditions—is essential.

### 8.3 Target-oriented aspects

The rapid clearance of EVs from circulation and their low uptake by vital tissues, such as the brain, heart, and tumors, indeed present a significant challenge for their clinical application in drug delivery (Lin et al., 2023). The short half-life of EVs in the bloodstream, often under 30 min, limits their therapeutic window and reduces their effectiveness (Lai et al., 2014; Wiklander et al., 2015). Additionally, the difficulty in tracking EVs *in vivo* complicates understanding their pharmacokinetics and increases concerns about their potential adverse effects (Zhao et al., 2024). To address this challenge, researchers are investigating alternative routes of administration and surface modifications on EVs to enhance their targeting properties, prevent rapid clearance by the circulatory system, and ensure more efficient delivery to diseased areas. Engineered modification of EV membrane proteins through genetic editing techniques represents an emerging strategy to enhance targeting specificity in EVs. Genetic editing techniques enable surface modification of EVs through the fusion of specific functional moieties (e.g., ligands or homing peptides) with transmembrane proteins, thereby enhancing their targeting precision and therapeutic efficacy (Yang et al., 2018). Liu et al. engineered EVs with surface-displayed PD-1 and the brain-targeting peptide angiopep-2 through protein modification, which significantly enhanced their targeting specificity (Liu et al., 2025). These modified EVs, co-loaded with Cas9 protein and multigene sgRNAs, implemented a minimally invasive gene editing approach that demonstrated marked antitumor effects in glioblastoma models. Moreover, light-controlled release represents an emerging strategy to enhance the targeting specificity of exosomes. Cheng et al. engineered an exosome-derived protein carrier capable of delivering distinct protein cargo to target cells (Cheng et al., 2021). Through genetic linkage with CD9-photocleavable fusion proteins, this system enabled light-controlled release of multifunctional payloads packaged within exosomes. These strategies aim to optimize EV biodistribution and amplify their therapeutic effects in specific target tissues.

Although significant progress has been made in the field of EVs targeting research, existing targeting strategies are still limited to temporarily increasing the concentration of EVs at the target site, which is largely attributed to the continuous diffusion of EVs into non-target tissues within the organism (Picon et al., 2023). In future clinical translation, integration of multiple EV-targeting strategies is expected to optimize the tissue distribution of EVs, thereby significantly enhancing therapeutic efficacy.

### 8.4 Preservation methods aspects

Another major challenge lies in the preservation of EVs. Cryogenic storage is commonly used for short-term preservation, but the process of long-term freezing or repeated freeze-thaw cycles can damage EVs, compromising their structure and functionality, ultimately affecting their therapeutic efficacy. Protective agents like glycerol, DMSO, and alginate can mitigate some damage, but careful control of their concentration and dosage is necessary (Görgens et al., 2022). Recently, new methods, such as dispersing EVs in concentrated hyaluronic acid solutions and transforming them into solid forms (EV microneedles), have shown promising results for long-term storage, maintaining EV stability for up to 6 months. This form also opens the possibility for oral administration, which could greatly expand the clinical applications of EVs(Bui et al., 2022). Improving EV preservation methods and optimizing their storage and handling protocols are indeed crucial areas of future research.

### 8.5 Regulation and ethics aspects

EVs-based therapies are generally safe and well tolerated, with a low overall incidence of adverse events, but more trials are needed to evaluate their long-term safety and efficacy (Van Delen et al., 2024). Although EVs have made breakthroughs in preclinical animal studies and several clinical trials have demonstrated the therapeutic role of EVs in various diseases, no EVs-based therapeutic drugs have been approved by national drug regulatory authorities for clinical practice (Bailey et al., 2022; Firouzabadi et al., 2024; Van Delen et al., 2024). In order for EVs-based disease treatments to benefit patients in the future, we need quantitative and transparent reporting, standardised protocols, reproducible Good Manufacturing Practice (GMP)-compliant manufacturing protocols, and the development of harmonised regulatory frameworks and policies (Gimona et al., 2017; Cheng, 2024). The application of GMP-compliant protocols to EV-based biotherapeutics development, when coupled with implementation of established regulatory requirements, would predictably lead to a near-term proliferation of methodologically robust clinical trials evaluating these therapeutic products. This advancement would significantly expedite the regulatory approval process for EVs as clinically validated therapeutic agents. In addition, a series of ethical issues regarding the origin, safety, privacy, and fairness of EVs as a vehicle for drug delivery are also among the challenges to be faced.

### 8.6 Pharmacokinetic aspects

Native EVs exhibit higher randomness in tissue distribution due to the lack of targeting ligands. Most studies indicate that after systemic administration, EVs tend to accumulate in organs such as the liver, spleen, and kidneys, while showing low active targeting efficiency toward therapeutic sites like the brain and tumors (Lázaro-Ibáñez et al., 2021). For example, high-sensitivity PET/MRI imaging detected only 0.5% of the injected dose of EVs in the brain following intravenous administration (Banerjee et al., 2019). Previous studies in mice, rats, and zebrafish models have demonstrated that EVs within the circulatory system possess a relatively short plasma half-life, generally not exceeding 30 min (Imai et al., 2015; Verweij et al., 2019; Wu et al., 2021). In contrast, a recent study using the non-human primate model of the pig-tailed macaque (*Macaca nemestrina*) showed that EV was consistently detected in plasma 24 h after administration by the intravenous and intranasal routes. This finding is much longer than the duration reported in mouse models (Driedonks et al., 2022). This suggests potential interspecies differences in EV clearance rates, highlighting the need to consider such variations in clinical applications. Elucidating EV distribution and clearance patterns can guide dosing frequency and dosage design to reduce unnecessary toxicity in non-target organs.

## 9 Conclusion

Over the past decade, EVs have advanced significantly as drug delivery vehicles. Collaborative efforts between medical and bioengineering disciplines have bolstered their drug-delivery capabilities, expanding their clinical potential. Extensive research has focused on optimizing the loading of various drug types—such as small molecules, proteins, and nucleic acids—into EVs, with each drug-loading method offering distinct advantages and limitations. Selection of EV sources, isolation techniques, and drug-loading strategies must be tailored to specific experimental needs. As preclinical studies progress, an increasing number of EV-based drug delivery systems have entered clinical trials, reflecting their growing applicability in areas like cancer therapy, neurodegenerative diseases, and immunotherapy. While challenges persist in large-scale production, targeting, storage stability, and clinical translation, ongoing efforts aim to refine manufacturing processes, enhance targeting strategies, and explore novel preservation techniques. EVs represent a promising frontier in drug delivery, poised to provide more effective tools for personalized therapy and precision medicine, and may become integral to future clinical practices.
